# Association of Clinical and Laboratory Parameters with Appendiceal Neoplasms in Acute Appendicitis: A Single-Institution Retrospective Cohort Study

**DOI:** 10.3390/medicina62081442

**Published:** 2026-07-24

**Authors:** Mia Krebs, Urška Kogovšek, Bojan Krebs

**Affiliations:** 1 Department of Diagnostic, Interventional and Pediatric Radiology (DIPR), Inselspital, Bern University Hospital, University of Bern, 3010 Bern, Switzerland; 2 Department of Abdominal Surgery, University Medical Center Ljubljana, Zaloška cesta 2, 1000 Ljubljana, Slovenia; 3 Department of Abdominal Surgery, University Medical Center Maribor, Ljubljanska ulica 5, 2000 Maribor, Slovenia

**Keywords:** appendiceal neoplasms, acute appendicitis, C-reactive protein, leukocyte count, clinical parameters, inflammatory parameters

## Abstract

*Background and Objectives:* Appendiceal neoplasms are frequently incidental findings in post-appendectomy histopathology, occurring in approximately 0.7–2.5% of patients with acute appendicitis. This study aimed to evaluate the association between clinical and laboratory parameters and the presence of appendiceal neoplasms in patients presenting with acute appendicitis. *Materials and Methods:* A retrospective cohort study was conducted, including patients treated at the University Medical Center Ljubljana between 2016 and 2020. Inclusion criteria were patients diagnosed with acute appendicitis who underwent emergency appendectomy, while patients with preoperatively diagnosed appendiceal neoplasms or non-surgical management were excluded. Patients with histopathologically confirmed appendiceal neoplasms were compared with a sample of patients without neoplasms, selected from the same cohort. Logistic regression analysis was performed to assess associations between clinical variables (age, sex, duration of symptoms, CRP, and leukocyte count) and neoplasm presence. *Results:* Appendiceal neoplasms were identified in 48 patients, corresponding to an analytic prevalence of 4.5% (48 of 1030) and a true prevalence of 1.6% (48 of 3014) among all appendicitis cases. In univariate analysis, age, duration of symptoms, CRP levels, and leukocyte count were significantly associated with neoplasm presence. In multiple logistic regression, higher CRP levels (OR 1.04, 95% CI 1.01–1.08; *p* = 0.007, per 10 mg/L increase) and lower leukocyte counts (OR 0.91, 95% CI 0.85–0.98, *p* = 0.012) remained significantly associated with appendiceal neoplasms. ROC analysis demonstrated limited discriminative performance (AUC < 0.70 for both parameters). *Conclusions:* Elevated CRP levels and lower leukocyte counts were associated with appendiceal neoplasms in patients presenting with acute appendicitis, suggesting a distinct inflammatory profile; however, their clinical utility for preoperative identification appears limited.

## 1. Introduction

Appendiceal neoplasms are rare and are typically identified incidentally in 0.7–2.5% of patients undergoing appendectomy [1,2,3]. They are broadly classified as epithelial or non-epithelial, with primary epithelial neoplasms and neuroendocrine tumors (NETs) the most common. Epithelial neoplasms include adenomas, low-grade mucinous neoplasms, high-grade mucinous neoplasms, mucinous adenocarcinomas, non-mucinous adenocarcinomas, and squamous cell carcinomas. NETs form a distinct group that includes classic and tubular subtypes. Less common appendiceal neoplasms include lymphomas, metastatic lesions, and mesenchymal tumors. Rare cases of non-carcinoid NETs, sarcomas, and neuroectodermal or nerve-sheath tumors have also been documented [4,5].

The clinical presentation of appendiceal neoplasms is heterogeneous and often nonspecific. Benign lesions may remain asymptomatic or be detected incidentally during appendectomy for presumed appendicitis, whereas malignant neoplasms can manifest through local invasion, peritoneal dissemination, or distant metastases [1,4,5]. The most frequent clinical presentation is acute appendicitis, reported in 30–50% of patients with appendiceal neoplasms [1,5,6]. In these cases, progressive neoplasm growth within the appendiceal lumen leads to obstruction, causing distension, venous congestion, and secondary infection. This process produces classic right lower-quadrant pain and inflammatory changes in the appendix. Obstruction typically occurs when neoplasms involve the body or base of the appendix, whereas lesions limited to the tip rarely cause luminal blockage [4].

Most patients with acute appendicitis undergo prompt surgical intervention, and all resected appendices are subjected to routine histopathological examination. The diagnosis of an appendiceal neoplasm is therefore usually established post-appendectomy, occasionally necessitating a second surgical procedure for definitive management. Surgical resection remains the cornerstone of treatment for the majority of appendiceal neoplasms [1]. Although most appendiceal neoplasms present clinically as acute appendicitis, their rarity makes a neoplastic etiology at appendectomy an infrequently considered possibility. Typically, the diagnosis must await histopathological examination of the appendiceal sample, as fewer than 50% of neoplasms are detected during or before surgery [7].

Currently, the only recognized and widely validated risk factors for appendiceal neoplasms are age and complicated appendicitis [8,9,10,11]. Other potential risk factors reported in the literature include female sex [11,12], American Society of Anesthesiologists (ASA) score [12], Crohn’s disease and immunosuppression/steroid use [13,14], absence of classic migratory right lower quadrant (RLQ) pain [10], and dynamics of inflammatory parameters [8,13,15,16,17,18]. However, these previously identified risk factors lack the sensitivity and specificity to be used in isolation for diagnosis [8]. Recent studies have focused on developing clinical risk scores to predict incidental appendiceal neoplasms in patients with acute appendicitis, particularly in older populations. These models combine clinical, laboratory, and imaging parameters; however, their overall predictive accuracy remains moderate. In this context, we investigated whether routinely available clinical and laboratory parameters are associated with appendiceal neoplasms in patients presenting with acute appendicitis.

## 2. Materials and Methods

This retrospective cohort study analyzed patient data from the Clinical Department of Abdominal Surgery, University Medical Center Ljubljana (UMC), Slovenia, for patients treated between January 2016 and December 2020. No significant changes in the diagnostic workup or management of acute appendicitis occurred during the study period.

### 2.1. Study Objectives

Primary objective: To evaluate the association between clinical and laboratory parameters, including age, sex, duration of symptoms, C-reactive protein (CRP) level, and leukocyte count, and the presence of appendiceal neoplasms in patients presenting with acute appendicitis.

Secondary objective: To describe the prevalence and histopathological distribution of appendiceal neoplasms among patients with acute appendicitis treated at a single tertiary care center.

### 2.2. Study Design

The study compared patients operated on for acute appendicitis with and without appendiceal neuoplasms. We reviewed demographic, medical, laboratory, operative, and histopathological data from all patients with acute appendicitis. Patients with histopathologically confirmed appendiceal neoplasms were then identified and compared with patients without neoplasms. The variables analyzed included duration of symptoms before diagnosis in the emergency department and inflammatory markers at the time of diagnosis, including C-reactive protein and leukocyte count.

### 2.3. Study Cohort

The study included patients treated in the Clinical Department of Abdominal Surgery at UMC Ljubljana between 2016 and 2020. Inclusion criteria were: (a) diagnosis of acute appendicitis at hospital admission and (b) emergency appendectomy as the primary operative procedure. Exclusion criteria were: (a) hospital treatment not primarily related to acute appendicitis, (b) appendectomy not performed as the initial emergency procedure, (c) appendectomy performed as part of another surgical procedure, (d) conservative treatment of appendicitis, and (e) appendiceal neoplasm diagnosed before surgery.

During the study period, 3014 patients without appendiceal neoplasms and 84 patients with histopathologically confirmed appendiceal neoplasms were identified. Among patients with appendiceal neoplasms, 36 were excluded because the neoplasm was discovered incidentally or in the context of another primary diagnosis, leaving 48 eligible patients with acute appendicitis treated by emergency appendectomy. Of these, 3 patients underwent appendectomy at another institution, and 1 patient had missing operative data, resulting in 44–48 evaluable patients depending on data availability.

**Figure 1:**
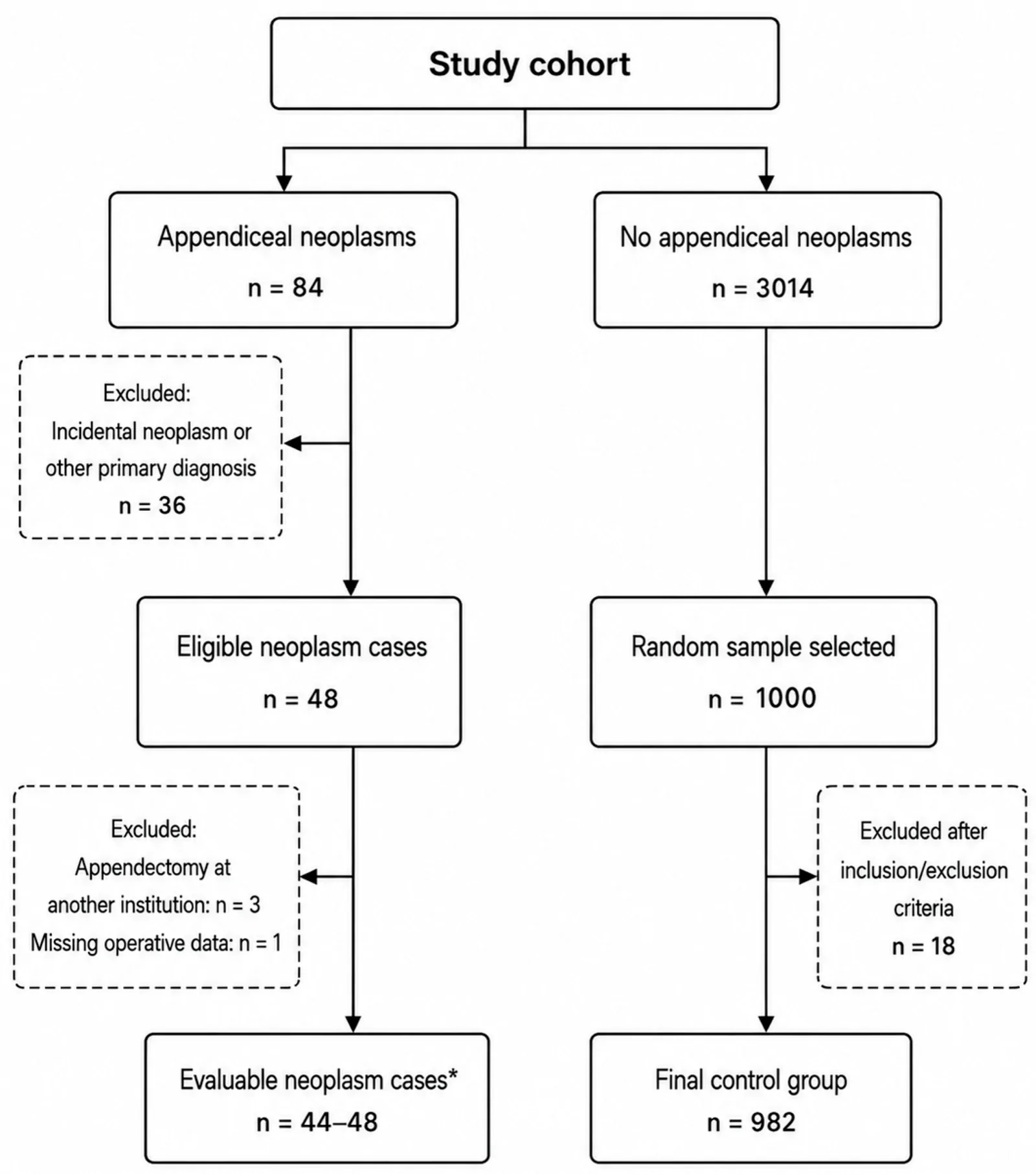
Flowchart of patient selection and study cohort formation. * Depending on data availability.

Because manual extraction of detailed clinical data from medical records was highly labor-intensive, a random sample of patients without appendiceal neoplasms was selected for detailed analysis. Simple random sampling without replacement was performed in R. To ensure reproducibility, a fixed random seed corresponding to the date of sample selection (2009–2021) was used. From the 3014 eligible patients without neoplasms, 1000 patients were randomly selected. After application of inclusion and exclusion criteria, 18 patients were excluded, leaving 982 patients in the control group. Among these, some variables contained missing data; therefore, analyses were performed using available-case data. A flowchart illustrating the patient cohort selection process is shown in Figure 1.

### 2.4. Data Collection

Data collection began by identifying patients treated at the Clinical Department of Abdominal Surgery, University Medical Center Ljubljana, between 2016 and 2020 with a diagnosis of acute appendicitis or appendiceal neoplasm. Hospitalization data were then manually extracted from the hospital database using these identification numbers. The dataset included demographic characteristics, medical history, laboratory results, radiological findings, operative details, and histopathological parameters. For patients with appendiceal neoplasms who underwent emergency appendectomy at another institution, data were obtained by contacting the relevant hospitals. Missing information from the electronic hospital database was supplemented, when available, with data from physical records stored in the UMC Ljubljana archive.

### 2.5. Statistical Analysis

Patients were stratified into two cohorts based on the presence or absence of an appendiceal neoplasm. Neoplasms were first categorized as benign or malignant based on histopathological and clinical characteristics. Benign lesions included benign polyps and low-grade mucinous neoplasms without disseminated disease, whereas malignant lesions included NETs, adenocarcinomas, and disseminated or histopathologically malignant mucinous neoplasms. However, because of the limited number of cases, all appendiceal neoplasms were analyzed collectively in subsequent regression analyses.

Intergroup differences in demographic and clinical characteristics were examined. Categorical variables are expressed as absolute frequencies and percentages. In contrast, continuous variables are reported as medians with interquartile ranges (IQRs) owing to significant deviations from normality, as verified by the Shapiro–Wilk test.

Associations between clinical variables and neoplasm presence were initially explored using univariate logistic regression. Variables demonstrating significant associations (*p* < 0.05) were subsequently entered into a multivariable logistic regression model to identify independent predictors, while minimizing model oversaturation given the relatively small neoplasm cohort. The multivariable model was adjusted for sex and age at diagnosis to assess the independent effects of symptom duration and inflammatory biomarkers (CRP and leukocyte count) on neoplasm occurrence. Owing to the limited number of neoplasm cases, the analysis was considered exploratory and hypothesis-generating rather than predictive. For improved clinical interpretability, CRP values were scaled per 10 mg/L increase in the multiple regression analysis.

Multicollinearity was assessed using variance inflation factors (VIFs), all of which ranged from 1.0 to 1.3, indicating no collinearity among covariates. Missing patient data were handled by available-case analysis. To evaluate potential selection bias associated with incomplete data, baseline characteristics of patients included in the multivariable model were compared with those excluded from the non-neoplasm group; no significant differences were observed in age at diagnosis (*p* = 0.693) or duration of hospitalization (*p* = 0.781).

All statistical computations were conducted using IBM SPSS Statistics, version 28.0 (IBM Corp., Armonk, NY, USA). Descriptive and inferential results were graphically summarized using quartile plots and visualized in Microsoft Excel 2022.

## 3. Results

### 3.1. Patient Demographic and Clinical Characteristics

Table 1 summarizes the demographic and clinical characteristics of the study population. The cohort included 1030 patients, of whom 501 (48.6%) were men. The median age at diagnosis was 36 years (IQR, 24–52), and the median time from symptom onset to diagnosis was 1 day (IQR, 0–2). The median duration of hospitalization was 2.65 days (IQR, 1.82–4.65). Median CRP was 32 mg/L (IQR, 9–92), and the median leukocyte count was 13.7 × 10^9^/L (IQR, 11–16.6). Appendiceal neoplasms were identified in 48 patients, corresponding to an analytic prevalence of 4.5% in the study sample and a true prevalence of 1.6% in the full cohort of 3014 patients with acute appendicitis. Of the neoplasm cases, 39 (81.3%) were malignant. NETs accounted for 52.1% of cases. All patients underwent emergency laparoscopic appendectomy at the initial operation, and 65% subsequently underwent elective laparoscopic right hemicolectomy after multidisciplinary team review.

### 3.2. Comparison of the Demographic and Clinical Characteristics of Groups of Patients with and Without Appendiceal Neoplasms

Table 2 presents the demographic and clinical characteristics of patients with and without appendiceal neoplasms, together with the results of the univariate logistic regression analysis. The presence of a neoplasm was significantly associated with age at diagnosis, time from symptom onset to diagnosis, CRP level, and leukocyte count. Patients with neoplasms were older, with a median age difference of 10 years between the two groups. For each additional year of age, the odds of having a neoplasm increased by 2%. A longer duration from symptom onset to diagnosis was also associated with neoplasm presence, with the odds increasing by 10% for each additional day. Higher CRP levels were associated with a greater likelihood of neoplasm presence, whereas higher leukocyte counts were associated with a lower likelihood of neoplasm presence.

### 3.3. Associations Between the Demographic and Clinical Characteristics of Patients and Neoplasm Presence

Table 3 presents the results of the multiple logistic regression. When the other independent variables in the model were controlled for, the CRP level and leukocyte count were significantly associated with the presence of neoplasms. For improved clinical interpretability, CRP values were scaled per 10 mg/L increase in the multivariable logistic regression model.

### 3.4. ROC Analysis

Table 4 presents the area under the receiver operating characteristic curve (AUC) for CRP, leukocyte count, and the multivariable model.

## 4. Discussion

The analysis showed differences between patients with acute appendicitis with and without appendiceal neoplasms in both clinical history and laboratory findings.

The results of the study are consistent with previously published data regarding the prevalence of appendiceal neoplasms. The analytic prevalence of appendiceal neoplasms was 4.5% (48 of 1030 patients), based on all neoplasm cases and a systematically selected random sample of non-neoplasm controls. The true prevalence in the entire appendicitis cohort was 1.6% (48 of 3014 patients), which aligns with rates reported in previous studies ranging from 0.7% to 2.5% [1,2,3]. The comparison group without neoplasms was selected from the full cohort using simple random sampling to reduce selection bias. Because no direct comparison with the full non-neoplasm cohort was performed, the analytic sample may be considered broadly comparable to the overall cohort, allowing only cautious generalization of the findings to patients treated during the study period. Of the detected neoplasms, 81.3% were malignant in this study, a slightly higher proportion than reported in other studies [8,13,19,20]. NETs had the highest prevalence among neoplasms in this study (52.1%), as in many other studies [8,9,19,21,22,23], a pattern that several factors can explain. The appendix contains a high density of neuroendocrine (enterochromaffin) cells, particularly at the tip, providing a large pool of cells of origin. In addition, NETs are typically small, well-differentiated, and clinically indolent, and are therefore most often detected incidentally during appendectomy [22]. Improved pathological examination and immunohistochemical techniques have further increased detection rates [24]. Finally, the peak incidence of NETs in younger patients coincides with the age group most frequently undergoing appendectomy, contributing to their higher observed prevalence [7].

Although the difference in neoplasm incidence between the sexes did not prove to be statistically significant, the results of other studies [12,13] show that appendiceal neoplasms are more common in women, and it is worth noting that in the cohort studied, neoplasms were present in 64.6% of women and only 34.5% of men. As NETs are the most common neoplasms in this study and studies suggest that they are slightly more common in females than in males [25,26], this could explain the difference in neoplasm incidence, but the evidence is still insufficient to conclude sex as a risk factor for appendiceal neoplasms.

Univariate analysis revealed significant associations between various parameters and the presence of an appendiceal neoplasm in patients with acute appendicitis. The likelihood of an appendiceal neoplasm increased with (a) advancing age, (b) longer duration from symptom onset to diagnosis, (c) higher CRP levels, and (d) lower leukocyte counts. Higher CRP levels and lower leukocyte counts remained significantly associated with a greater likelihood of neoplasm, even after controlling for other independent covariates in multiple logistic regression. These findings suggest a distinct inflammatory profile in patients with appendiceal neoplasms.

Several studies support the observation that lower leukocyte counts and higher CRP levels are associated with appendiceal neoplasms. A large analysis using the ACS-NSQIP database demonstrated that a lower preoperative white blood cell (WBC) count was an independent predictor of appendiceal neoplasm, with the odds of neoplasm pathology increasing by approximately 3% for each one-unit decrease in WBC count on multivariable analysis [13]. Similarly, in a propensity score-matched cohort of 5829 patients, neutrophil count was identified as an independent predictor of appendiceal neoplasia [16].

Several mechanisms may explain these findings. Acute appendicitis is typically characterized by a rapid and pronounced neutrophilic leukocytosis driven by bacterial invasion and luminal obstruction. In contrast, appendiceal neoplasms often cause gradual luminal obstruction without acute bacterial translocation. The resulting inflammatory response is more chronic and less intense, leading to relatively lower leukocyte counts. In addition, certain neoplasm types, particularly mucinous neoplasms, may produce a low-grade, smoldering inflammatory process that does not elicit a marked leukocytic response [8,13]. CRP, as an acute-phase reactant regulated by cytokines such as interleukin-6, reflects both acute and chronic inflammation. In appendiceal neoplasms, CRP elevation may be explained by several mechanisms. First, neoplasm-associated inflammation leads to persistent cytokine release, promoting hepatic CRP production. Second, neoplasms are more frequently associated with complicated appendicitis, including perforation, abscess, or phlegmon formation, which are known to markedly increase CRP levels [8,15,17].

The differing kinetics of inflammatory markers may further explain this discrepancy. Leukocyte counts typically rise early in the inflammatory process and may plateau or normalize, whereas CRP levels increase progressively over time. Patients with appendiceal neoplasms often present with a longer duration of symptoms, allowing for continued CRP elevation despite less pronounced leukocytosis [13,18].

The combination of relatively lower leukocyte counts and disproportionately elevated CRP levels may therefore represent a distinct inflammatory pattern in neoplasm-associated appendicitis. In typical acute appendicitis, WBC and CRP tend to rise in parallel; thus, a discordant pattern may raise suspicion for an underlying neoplasm. However, it should be emphasized that this pattern does not apply uniformly across all neoplasm subtypes. NETs, particularly small and well-differentiated lesions, often do not demonstrate significant differences in clinical, laboratory, or radiological parameters compared with benign appendicitis. Consequently, the observed associations of lower leukocyte counts and higher CRP levels are more likely to reflect aggressive epithelial malignancies, such as adenocarcinoma, high-grade mucinous neoplasms, or goblet cell carcinoma, rather than indolent NETs [22]. Because all neoplasm subtypes were analyzed together, the observed associations likely represent overall patterns across a biologically heterogeneous group rather than subtype-specific effects. This is important because NETs, mucinous neoplasms, adenocarcinomas, and benign polyps may differ substantially in their inflammatory profiles and clinical presentation.

Although age and time from symptom onset to surgery were not statistically significant in the multiple logistic regression, other studies have shown that these variables are significantly associated with a greater likelihood of an appendiceal neoplasm in acute appendicitis patients. Age is the most robust and consistently validated clinical predictor of appendiceal neoplasms across multiple studies. The incidence of neoplasms increases progressively with age, and various age thresholds have been proposed, with ≥40 years associated with a significantly increased risk, particularly when combined with imaging findings such as an appendiceal diameter > 10 mm [10,21,27,28]. The incidence of appendiceal neoplasms is increasing, and owing to the progressive aging of the population, an increase in the number of cases of acute appendicitis in older adults has been reported [1,20]. On the other hand, treatment patterns for acute appendicitis are also changing. In recent years, several studies have demonstrated the safety and efficacy of nonoperative management of acute appendicitis in selected patient populations. Consequently, this approach is increasingly being adopted in clinical practice, although the potential long-term implications related to underlying appendiceal neoplasms may not always be fully considered. Therefore, both during evaluation for conservative management and before emergency appendectomy, the increased risk of appendiceal neoplasms in older patients should be taken into account.

A longer duration from symptom onset to diagnosis was observed in patients with appendiceal neoplasms in the univariate analysis, consistent with findings from previous studies [22,29]. This may be explained by the typically indolent growth pattern of appendiceal neoplasms, which can cause intermittent or partial luminal obstruction rather than an acute inflammatory process. As a result, symptoms may develop gradually and remain relatively mild or nonspecific over time. Patients may therefore adapt to these symptoms or manage them conservatively, leading to delayed presentation and diagnosis compared to cases of acute appendicitis of non-neoplastic origin.

It is noteworthy that the significant associations of age and symptom duration observed in the univariate analysis were no longer present in the multivariable logistic regression model, which included sex, age, time from symptom onset to diagnosis, CRP level, and leukocyte count. This may suggest that inflammatory parameters, particularly CRP and leukocyte count, are more strongly associated with appendiceal neoplasms in the preoperative setting. Alternatively, the loss of significance for age and symptom duration may reflect collinearity or interaction between variables, with their effects partly mediated through inflammatory markers. It is important to note that the relatively small number of neoplasm cases may have reduced statistical power and limited the stability and discriminative performance of the multivariable model.

Although the multivariable model demonstrated modest discrimination, the observed AUC values suggest only limited clinical utility for preoperative decision-making. These findings, therefore, should be interpreted primarily as exploratory associations rather than as evidence of a clinically applicable prediction model. In particular, the results indicate that CRP and leukocyte count may reflect differences between the two patient groups. Still, they do not appear sufficiently robust to support reliable individual-level classification before surgery.

### Strengths and Limitations

This study has several strengths. First, most patients with acute appendicitis in Slovenia undergo surgery at UMC Ljubljana, where the majority of appendiceal neoplasms are also diagnosed. In combination with the centralized management of patients with appendiceal neoplasms in Slovenia, this provided a relatively large study cohort. The use of population-based data also improves the generalizability of the findings compared with single-institution studies with smaller patient numbers. In addition, because the study was conducted at a single institution, patients were managed according to a standardized protocol, which supports consistency in data collection and clinical care.

This study has several limitations. Its retrospective, single-center design may limit the external validity and generalizability of the findings. In addition, using a sampled control group rather than the full non-neoplasm cohort introduces the possibility of selection bias, despite systematic sampling. The relatively small number of patients with appendiceal neoplasms limits statistical power and may affect the robustness of the multivariable regression model. With a limited number of outcome events and several predictors, overfitting and wide confidence intervals are possible; therefore, the findings should be interpreted as exploratory rather than confirmatory. Data accuracy depends on the completeness and reliability of electronic and written medical records, which can introduce information bias. Laboratory measurements were performed in multiple laboratories, which may have introduced inter-laboratory variability. The reported duration of symptoms was based on patient-reported information and is therefore subject to recall bias and variation in symptom perception. The neoplasm group was also heterogeneous, including multiple histological subtypes, which may have influenced inflammatory profiles and weakened subtype-specific associations. Relevant confounders, including complicated appendicitis, perforation, abscess, appendiceal diameter, and imaging findings, were not consistently available and were therefore excluded from the analysis. Their absence may have led to residual confounding, as these factors could also influence inflammatory markers and be associated with neoplasm presence.

## 5. Conclusions

Patients with appendiceal neoplasms presenting as acute appendicitis demonstrated a distinct inflammatory profile characterized by elevated CRP levels and lower leukocyte counts compared with patients without neoplasms. Although these laboratory parameters were independently associated with neoplasm presence, their discriminative performance was limited and does not support their use as reliable markers for preoperative identification of appendiceal neoplasms. Nevertheless, recognition of these associations may increase clinical awareness, particularly in the context of the increasing use of nonoperative management strategies for acute appendicitis, where underlying neoplasms may otherwise remain undetected.

Given the heterogeneous spectrum and increasing incidence of appendiceal neoplasms, further large-scale prospective multicenter studies incorporating clinical, laboratory, and radiological variables are warranted to better characterize their preoperative presentation and to evaluate their potential role in risk stratification and individualized management strategies.

## Figures and Tables

**Table 1:** Baseline characteristics of the study cohort.

Characteristic	*n* = 1030
Male sex	501 (48.6)
Age at diagnosis, years	36 (24–52; 1029)
Time to diagnosis, days	1 (0–2; 1020)
Time of hospitalization, days	2.65 (1.82–4.65; 1029)
CRP, mg/L	32 (9–92; 1001)
Leukocytes, ×10^9^/L	13.7 (11–16.6; 996)
Neoplasm	48 (4.7)
Malignancy	39 (81.3)
**Histology**	
Malignant low-grade mucinous neoplasm	4 (8.3)
Benign low-grade mucinous neoplasm	3 (6.3)
Neuroendocrine tumors	25 (52.1)
Adenocarcinoma	12 (25.0)
Benign polyp	6 (12.5)
**1. surgical procedure**	
Urgent laparoscopic appendectomy, *n* (%)	48 (100)
**2. surgical procedure (*n* = 18)**	
Elective laparoscopic right hemicolectomy, *n* (%)	13 (72)
Elective robotic right hemicolectomy, *n* (%)	5 (28)

Values are presented as *n* (%) or median (IQR; *n*), unless otherwise indicated. CRP, C-reactive protein; IQR, interquartile range.

**Table 2:** Univariate analysis of factors associated with appendiceal neoplasms.

Characteristic	No Neoplasm (*n* = 982)	Neoplasm (*n* = 48)	OR (95% CI)	*p*-Value
Male sex	484 (49.3)	17 (35.4)	0.56 (0.31–1.03)	0.064
Age at diagnosis, years	36 (24–51; 982)	46 (33–58; 47)	1.02 (1.01–1.04)	0.004
Time to diagnosis, days	1 (0–2; 973)	2 (1–3; 47)	1.10 (1.01–1.18)	0.021
CRP, mg/L	32 (9–88; 954)	75 (28–170; 47)	1.01 (1.00–1.01)	<0.001
Leukocyte count, ×10^9^/L	13.7 (11.1–16.6; 949)	12.1 (9.5–14.6; 47)	0.91 (0.85–0.98)	0.016
Time of hospitalization, days	2.62 (1.81–4.60; 982)	3.00 (2.01–5.88; 47)	1.01 (0.96–1.07)	0.656

Values are presented as *n* (%) or median (IQR; *n*), unless otherwise stated. OR, odds ratio; CI, confidence interval; CRP, C-reactive protein; IQR, interquartile range.

**Table 3:** Multivariable analysis of factors associated with appendiceal neoplasms.

Characteristic	OR (95% CI)	*p*-Value
Male sex	0.60 (0.32–1.12)	0.11
Age at diagnosis, years	1.02 (1.00–1.03)	0.072
Time to diagnosis, days	1.05 (0.96–1.16)	0.263
CRP, per 10 mg/L	1.04 (1.01–1.08)	0.007
Leukocyte count, ×10^9^/L	0.91 (0.85–0.98)	0.012

OR, odds ratio; CI, confidence interval; CRP, C-reactive protein.

**Table 4:** ROC analysis of inflammatory markers and the multivariable model.

Variable	AUC	95% CI
CRP	0.65	0.57–0.73
Leukocyte count	0.61	0.53–0.70
Multivariable model	0.72	0.64–0.79

AUC, area under the receiver operating characteristic curve; CI, confidence interval; CRP, C-reactive protein.

## Data Availability

The datasets used and/or analyzed during the current study are available from the corresponding author upon reasonable request.

## Abbreviations

The following abbreviations are used in this manuscript:

AUC	Area Under the Curve
CI	Confidence Interval
CRP	C-reactive Protein
IQR	Interquartile Range
NET	Neuroendocrine Tumor
RLQ	Right Lower Quadrant
UMC	University Medical Center
WBC	White Blood Cell
